# The Complement C3a and C3a Receptor Pathway in Kidney Diseases

**DOI:** 10.3389/fimmu.2020.01875

**Published:** 2020-08-18

**Authors:** Shuang Gao, Zhao Cui, Ming-hui Zhao

**Affiliations:** ^1^Renal Division, Peking University First Hospital, Beijing, China; ^2^Institute of Nephrology, Peking University, Beijing, China; ^3^Key Laboratory of Renal Disease, Ministry of Health of China, Beijing, China; ^4^Key Laboratory of CKD Prevention and Treatment, Ministry of Education of China, Beijing, China; ^5^Peking-Tsinghua Center for Life Sciences, Beijing, China

**Keywords:** complement, C3a, C3a receptor, kidney disease, inflammation

## Abstract

The pathogenesis of some kidney diseases is closely associated with complement activation, where the C3a/C3a receptor (C3aR) might play a crucial role. C3a/C3aR has dual roles and may exert anti-inflammatory or pro-inflammatory effects depending on different cell types and diseases. In the kidneys, C3aR is primarily expressed on the tubular epithelium and less in glomerular podocytes. C3aR expression is enhanced and the levels of C3a in the plasma and urine are increased in kidney diseases of several types, and are associated with disease progression and severity. The C3a/C3aR pathway facilitates the progression of glomerular and tubulointerstitial diseases, while it has opposite effects on urinary tract infections. Clinical trials targeting C3a/C3aR in kidney diseases are lacking. Here, we reviewed the studies on the C3a/C3aR pathway in kidney disease, with the aim of understanding in-depth its controversial roles and its potential therapeutic value.

## Introduction

**Figure 1:**
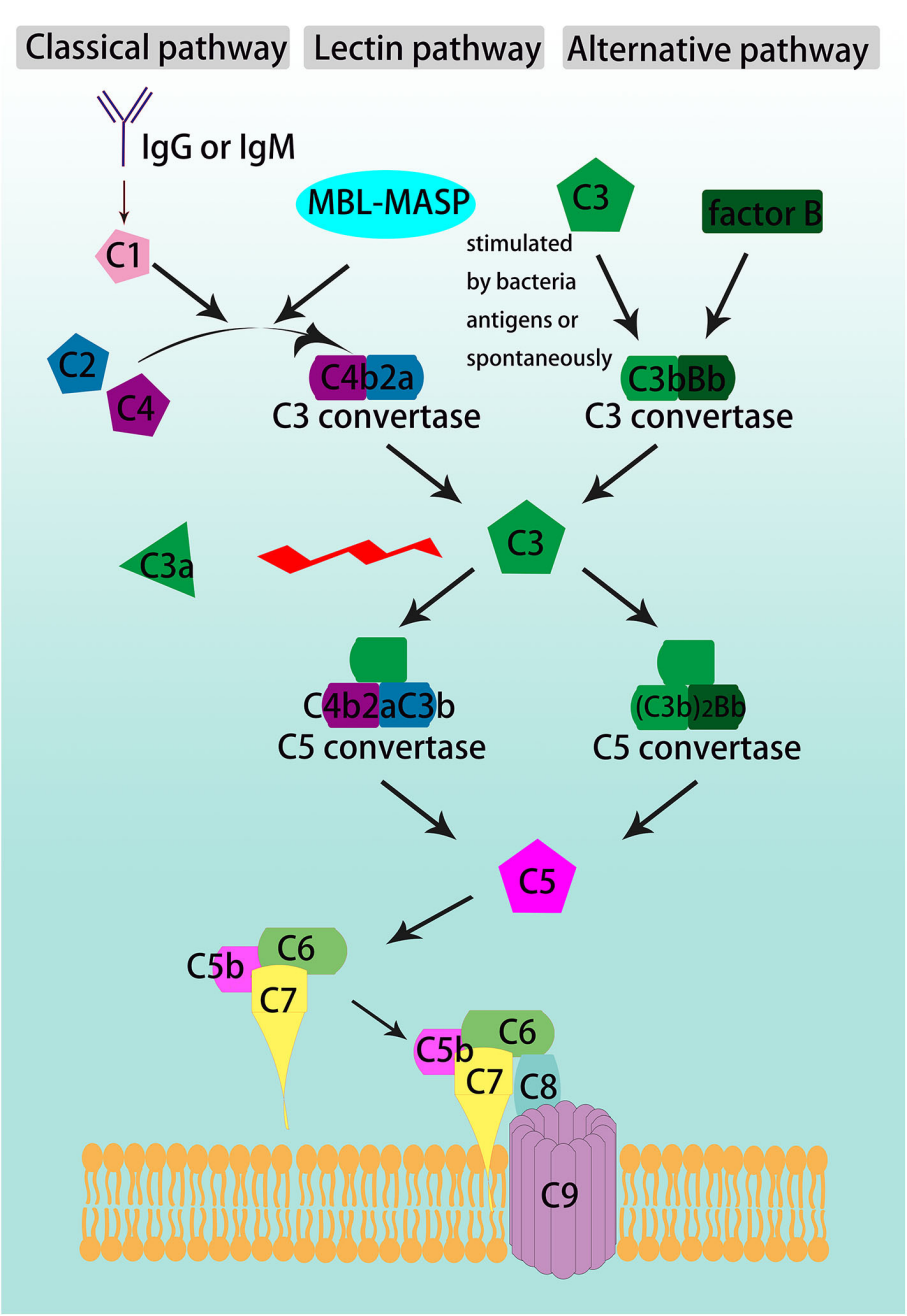
Schematic overview of complement cascade. The complement system is activated through the classical, lectin, and alternative pathways, converge at the formation of C3 convertases. The classical pathway is activated by either IgG or IgM, the lectin pathway is triggered by the binding of mannose-binding lectin (MBL) to the pathogens' polysaccharide surface, and the alternative pathway begins with C3 spontaneous cleavage. C3 is the beginner and the core member of common pathway which is formed by the convergence of the three initiating pathways and activate C3, C5-C9 through sequence to form the terminal complement cascade, namely membrane attack complex (MAC).

Complement activation participates in the pathogenesis of a variety of diseases and induces tissue damage. The complement system is activated through three pathways (Figure 1). The classical pathway is initiated by the interaction of C1 with IgG or IgM antibodies, and then by cleavage of C2 and C4 to form the C3 convertase, C4b2a. In the lectin pathway, mannose-binding lectin (MBL) binds to the bacterial polysaccharide surface to form a complex, which also cleaves C2 and C4 to generate C4b2a. The alternative pathway involves spontaneous activation, C3 is cleaved to generate C3b, then C3b constitutes another C3 convertase, C3bBb, with factor B (1).

The three pathways converge on C3 convertase, C4b2a or C3bBb, which cleaves C3 and begins the common pathway. C3 is split between arginine (Arg) 77 and serine (Ser) 78 into two fragments, the smaller one as C3a and the larger one as C3b (2, 3). C3b is an opsonin that can lead to destruction of microbes by coating and decorating them. It also participates in the common pathway by forming the C5 convertase, C4b2aC3b (in classical and lectin pathways) and (C3b)_2_Bb (in alternative pathway). C5 convertase cleaves C5 into C5a and C5b. C5b binds to C6 and C7 to form a trimeric complex. Then, C8 and C9 bind to form the membrane attack complex (MAC). The lipophilic protein C7 makes the complex attached to the cell membrane by inserting into the lipid bilayer (2).

C3a is an anaphylatoxin that exerts various effects after binding to the C3a receptor (C3aR). In kidneys, C3aR is expressed at high levels on tubular epithelial cells (4). In glomeruli, the staining for C3aR is restricted to glomerular epithelial cells (podocytes) and is much weaker than that in the tubule (4). There are controversies about the roles of C3a/C3aR in kidney disease onset and tissue damage. In this review, we focused on the inflammatory effects after C3a binding with C3aR and its roles in the development of kidney diseases.

### C3a and C3aR

Human C3a is a small 9 kDa peptide that comprises 77 amino acids and has four anti-parallel helical structures that are trapped by three disulfide bridges (5). The binding site of C3a to C3aR includes the C-terminal amino acids leucine-glycine-leucine-alanine-Arg (3, 5). When C3a is cleaved to C3a-desArg, it loses the ability to bind to C3aR, but binds to the second receptor of C5a, named C5aR2 (5). The antibodies currently used to detect C3a can distinguish C3 from its cleavage product; however, they fail to discriminate C3a from C3a-desArg, and may even detect C3a-desArg with higher sensitivity (5). Thus, it is necessary to develop antibodies that specifically recognize C3a. In healthy individuals, the plasma concentration of C3a is 119 ng/ml, and is derived from the degradation of C3 in the alternative pathway (5). However, the C3a concentration varies widely in different studies, from 20 to 156 ng/ml, due to the protocol and sample characteristics. The levels of C3a are increased in the sera due to removal of proteases that hydrolyzes C3a in the process of plasma clotting. Studies have reported that C3a levels in the circulation were elevated in pregnant women and may reach 182.5 ± 150.0 ng/ml in the plasma in the first trimester (5, 6).

Human C3aR is a 55 kDa protein comprising 482 amino acids and is a seven-transmembrane domain receptor that belongs to the G-protein coupled receptor family (4, 7–9). C3aR was first cloned in 1996 and was isolated from a cDNA expression library from U-937 cells (8). The human C3aR gene is located on chromosome 12p13.2-3 as a single copy. C3aR mRNA expression has been detected in several major organs such as the kidney (4), brain (10), lung (11), intestine (12), subcutaneous adipose tissue (13), and other (9). There are mainly four post-translational modifications (14): C3aR is highly glycosylated; tyrosine sulfated (9, 15) and especially Tyr174 plays a critical role in high-affinity binding to C3a; phosphorylated and S-acylated (5). After binding to C3a, C3aR is activated and triggers intracellular signaling. The principle signaling pathway is mediated by the pertussis toxin (PT)-sensitive G protein Gα_i_ in immune cells (16) and the PT-insensitive Gα12/13 that leads to the activation of the ERK1/2 pathway and cytoskeletal changes (17). C3aR activation also causes an increase in intracellular Ca^2+^, while C5aR activation has a stronger effect on promoting Ca^2+^ elevation (5). Another essential mediator is arrestin that can terminate the influence of C3aR to Ca^2+^. A C3aR mutant failed to bind to β-arrestin 2. Thus, in mast cells, C3aR desensitization and internalization were inhibited by silencing β-arrestin 2, which leads to a prolonged release of Ca^2+^ (18).

### The Function of C3a/C3aR in the Immune System

**Figure 2:**
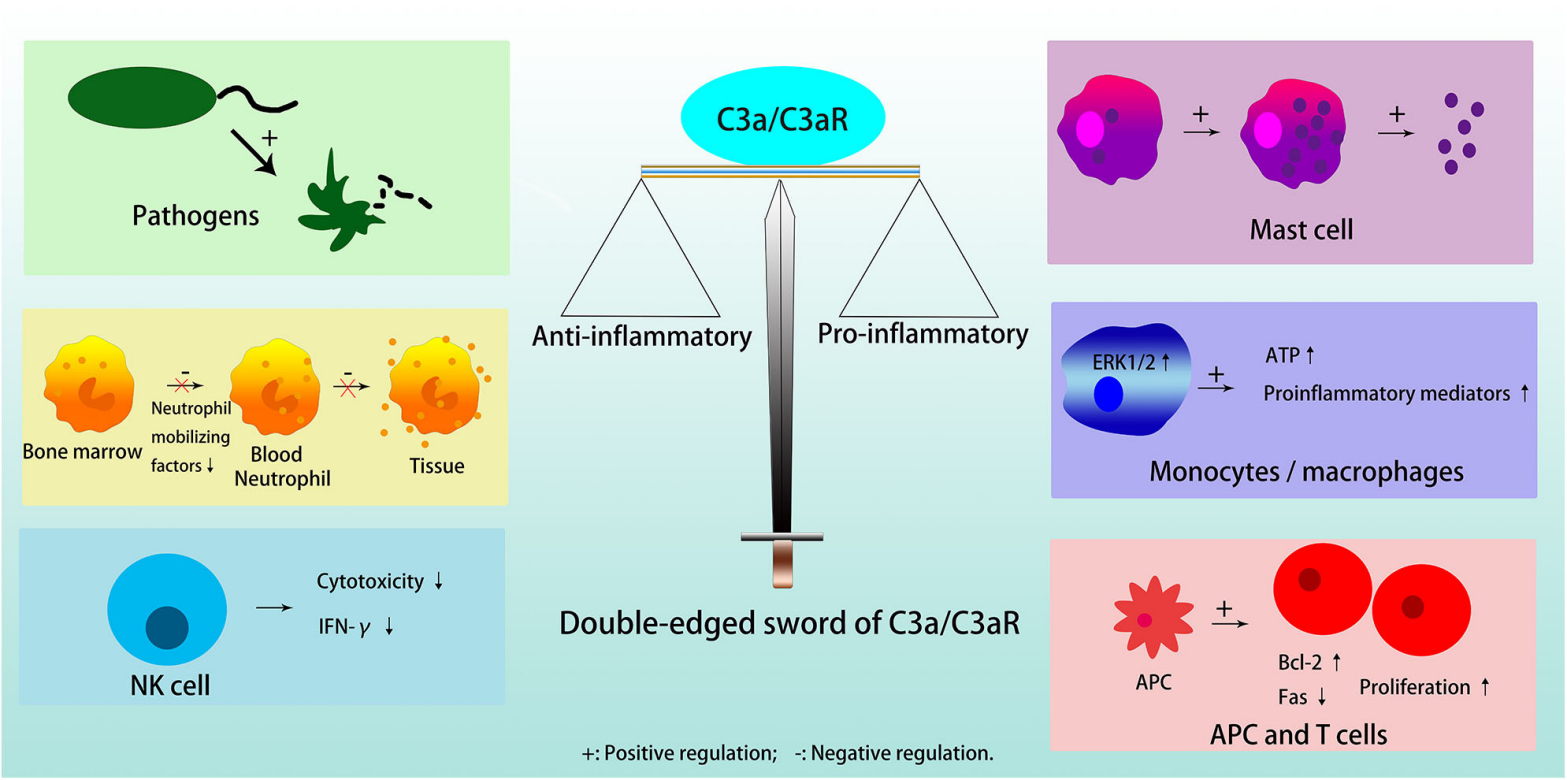
Double edged sword of C3a/C3aR. C3a/C3aR plays anti-inflammatory effects in inducing pathogens elimination, inhibiting neutrophil migration from bone marrow and degranulation in tissue, and reducing cytotoxicity and expression of IFN-γ in NK cell. C3a/C3aR plays pro-inflammatory effects in inducing mast cells to generate and secrete small granule spherical particles, upregulating the expression of proinflammatory mediators by activating ERK1/2 and releasing extracellular ATP in monocytes or macrophages, and promoting T cell proliferation and differentiation directly or indirectly through APC.

**Figure 3:**
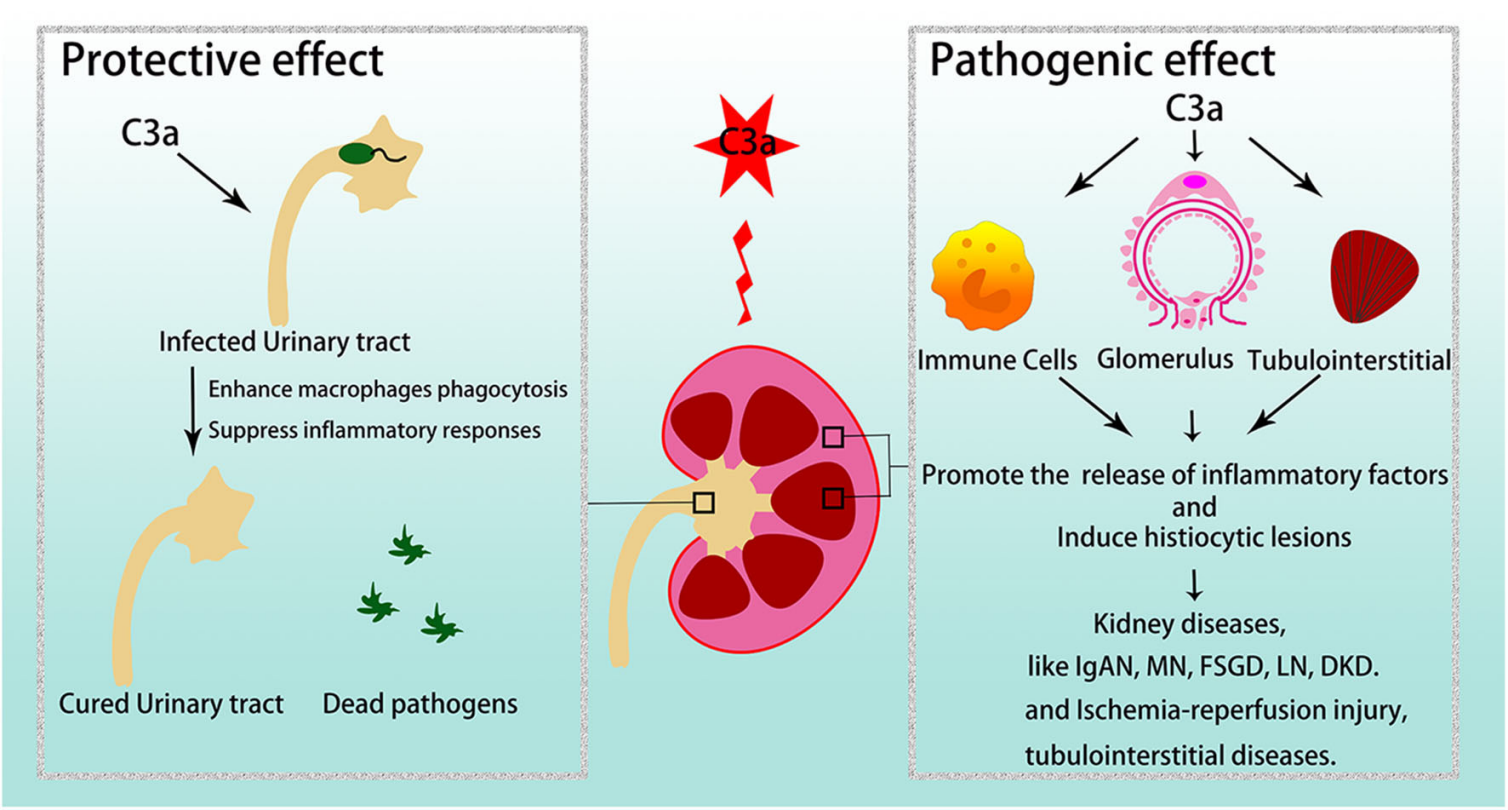
C3a/C3aR in kidney diseases. C3a/C3aR is related to disease development and severity in various glomerular diseases and tubulointerstitial injuries. However, in urinary tract infections, C3a/C3aR acts protective effect. IgAN, IgA nephropathy; MN, membranous nephropathy; FSGS, focal segmental glomerulosclerosis; LN, lupus nephritis; DKD, diabetic kidney disease.

C3a is a critical chemotactic mediator in the immune system, and C3a/C3aR pathway has a dual, anti-inflammatory and pro-inflammatory roles in different cells and diseases [Figure 2; (3)]. In general, C3a/C3aR participates in the response of the immune system in three ways, acting on resident innate immune cells to up-regulate or down-regulate different cytokines, activate dendritic cells (DCs), and regulate T cell signaling between lymphocytes and antigen presenting cells (APCs) (19).

C3a acts anti-inflammatory effects in pathogens, i.e., bacteria/fungi, neutrophils in the bone marrow reservoir, and natural killer (NK) cells. Furthermore, although there is a high expression of functional C3aR on the neutrophil cell membrane, C3a does not stimulate neutrophil degranulation to activate inflammatory response (3). C3a can also inhibit the migration of neutrophils from the bone marrow into the circulation by directly inhibiting neutrophil mobilizing factors, for example, G-CSF (3, 18). In NK cells, C3a is a negative regulator because it is able to not only inhibit NK cell cytotoxicity *in vivo*, but also to down-regulate the expression of IFN-γ (20).

C3a exerts pro-inflammatory functions in mast cells, macrophages/monocytes, T cells, and APC. Mast cells participate in allergic reactions, in which anaphylatoxin C3a induces them to produce and secrete small granule spherical particles, in a process named IgE-independent degranulation, through a rapid rise of intracellular calcium levels (21, 22). In monocytes or macrophages, C3a induces the release of proinflammatory mediators after binding to C3aR, activation of ERK1/2, and release of extracellular ATP, which in turn induces P2X7 that cooperates with NF-κB and enhances the production of IL-1β (23). When the activity of monocytes prevails over neutrophils, C3a plays a catalytic role in inflammation in general (3). In T cells, C3a/C3aR activates phosphoinositide-3-kinase-γ and induces phosphorylation of AKT, while, it up-regulates the antiapoptotic protein Bcl-2 and down-regulates the proapoptotic molecule Fas, to decrease T cell apoptosis and enhance their proliferation (24). In addition, C3a/C3aR can promote T cell proliferation through its effect on APC (23). The absence of C3, C3a or C3aR on APC results in a reduction in MHC II expression, which limits T cell proliferation and differentiation by insufficient antigen presentation (25, 26). C3aR blockade or deficiency results in decreased secretion of inflammatory factors, such as IL-2 and IFN-γ in T cells, and IL-1, IL-12, and IL-23 in APC (26).

In the studies of C3a and C3aR, the C3aR agonists and antagonists play pivotal roles. The peptide ligand WWGKKYRASKLGL, also called “super agonist,” is the most effective C3aR agonist, 15-fold more potent than C3a. There are several new potent and selective agonists validated by the calcium release assay, that is, FLPLAR 26/24 and FWTLAR 54/55. The most common and effective C3aR antagonist SB290157 is a trifluoroacetate salt, with an effective IC_50_ of 27.7, 7.0, and 12.5 nM in human, mouse, and guinea pig RBL-2H3 cells, respectively (27). Nevertheless, SB290157 treatment had off-target activity resulting in rapid neutropenia and transient hypertension (28). There are even reports suggesting that SB290157 functions as an agonist. C3a binds to C3aR to induce calcium mobilization, a marker of C3aR activation, whereas SB290157 also induced calcium mobilization in a dose-dependent manner (28). SB290157 showed antagonist effects on cells with low levels of C3aR expression, while it acted as an agonist on cells with high levels of C3aR (29).

C3aR knockout animals have been employed in numerous experiments, and questions arise regarding the choice of complete knockout or conditional knockout. In complete knockout animals, both local organs studied and circulating immune cells lack C3aR expression. Considering the different functions of C3aR in different cells, its role in circulating cells might interfere with the effects on local organs. The conditional knockout animals can restrict C3aR deficiency in specific organs, but the circulating immune cells that participate in the pathogenesis still possess normal expression of C3aR, which may weaken the effects. Thus, cautious interpretations should be given when there is a difference between the results from knockout animals and those with the C3aR antagonist.

C3a/C3aR participates in the pathogenesis of various diseases. In Alzheimer's disease, the C3a/C3aR pathway mediated microtubule-associated protein tau modulation by targeting STAT3, and the expression of C3aR was negatively correlated with cognitive function and positively correlated with Braak stages (10). In asthma, C3a/C3aR promoted smooth muscle contraction, mucus secretion, and recruitment of inflammatory cells. Deficiency of C3aR was protective to the lungs in a murine model of allergic airway disease (30, 31). In coronary artery disease, the expression of C3aR was positively correlated with activated glycoprotein IIb/IIIa in platelets, and the incidence of stroke and myocardial infarction was reduced in C3aR^−/−^ mice (32). C3a/C3aR played a protective role in intestinal ischemia-reperfusion (IR) injury by inhibiting neutrophil mobilization (33).

### C3a/C3aR in Kidney Diseases

C3a/C3aR plays crucial roles in various kidney diseases (Figure 3).

#### Primary Glomerular Diseases

##### IgA nephropathy (IgAN)

IgAN is characterized by mesangial IgA and C3 deposition, and C3a/C3aR contributes to its pathogenesis. In 1992, Abou-Ragheb et al. found a positive correlation between plasma C3a levels and plasma creatinine levels in patients with IgAN, and suggested the measurement of plasma anaphylatoxins as a possible indicator of disease activity and prognosis (34). However, Janssen et al. found that the C3a levels could not predict renal prognosis or reflect the status of neoantigens that develop after C3 activation, which is an indicator of disease activity and outcome (35). In 2014, Liu et al. observed elevated staining of C3aR and C3a in glomeruli, and high levels of C3a in the sera and urine from IgAN patients (36). Both urinary C3a levels and glomerular C3aR and C3a staining correlated positively with proteinuria, serum creatinine levels, and histopathological injuries (30). In 2015, Zhu et al. found associations of variants of genes encoding complement factor H (CFH), CFH-related protein 3 (CFHR3), CFH-related protein 1 (CFHR1) with high levels of circulating C3 and low-levels of serum C3a in IgAN patients. CFHR1 protein is a competitive antagonist of CFH; therefore, higher levels of CFH inhibit complement activation, leading to the inhibition of C3 cleavage to C3a. Thus, in patients with low CFH, CFHR1, or CFHR3, there is excess cleavage of C3 and a buildup of C3a in the plasma (37). In 2016, a study of a cohort of patients with IgAN revealed that plasmapheresis is effective in reducing urinary C3a levels and the probability of dialysis-dependence (38). In 2017, Zhang et al. showed that C3aR knockout mice had lower levels of proteinuria and reduced IgA and C3 deposition in the kidney as well as reduced histological injury when IgAN was induced by Sendai virus. They also detected a decrease in TNF-α, TGF-β, IL-1β, IL-6, and monocyte chemoattractant protein-1 (MCP-1) in mouse kidneys (39). In 2019, Zhang et al. found that plasma C3a in patients with IgAN was positively correlated with plasma and mesangial galactose-deficient IgA1 molecules (Gd-IgA1) (38, 40).

These studies suggest a direct role of C3a/C3aR in IgAN, although it is unclear whether its involvement is at the level of the kidney. A conditional knockout mouse, where C3aR is deleted from the kidney, will be a useful tool to elucidate this.

##### Membranous nephropathy (MN)

Primary MN is currently identified as an autoimmune disorder of M-type phospholipase A2 receptor (PLA2R), which is expressed on podocyte membrane. Cross-sectional studies have found that C3a levels were remarkably high in the plasma and urine of MN patients, and they were even higher in the circulation of patients with positive total and IgG4 anti-PLA2R antibodies than those without the antibody. Patients who achieved complete remission had lower levels of serum C3a (41, 42). The deposition of IgG4 anti-PLA2R is a reliable predictor of MN that activates the complement cascade by binding to MASP-1/2/MBL complex (42). In hepatitis B virus-associated MN, the significant reduction in plasma C3a levels was also related to remission during treatment (43).

We can speculate that C3a/C3aR is involved in MN pathogenesis. Previous studies have only shown that circulating C3a levels increase and much work is needed to confirm the role of the C3a/C3aR pathway. For example, we can further investigate C3aR expression in podocytes, and can clarify the pathogenic role of C3a/C3aR by inducing disease in C3a/C3aR deficient animals or by blocking C3aR.

##### Focal segmental glomerulosclerosis (FSGS)

FSGS is caused by the loss or injury of podocytes. In 2016, Morigi et al. found that C3a/C3aR participated in podocyte depletion and glomerulosclerosis. In FSGS patients with progressive proteinuria, protein-overload mice and *in vitro* podocyte culture, C3a/C3aR caused podocyte damage by activating glial cell line-derived neurotrophic factor (GDNF)/c-Ret (the receptor of GDNF) pathway, which is a critical adaptive response when podocytes are exposed to toxic injury. C3a/C3aR also induced parietal epithelial cells to up-regulate CXCR4, which resulted in parietal epithelial cell proliferation and migration, and glomerulosclerosis (44). In adriamycin (ADR)-treated mice, Liu et al. found that C3aR expression was increased in the kidneys. Furthermore, it has been found that resveratrol suppressed inflammatory response, glomerulosclerosis, and renal interstitial fibrosis through down-regulation of the C3aR/C5aR-Sphk1 pathway (33).

C3a/C3aR also plays a role in tubulointerstitial fibrosis in FSGS. In 2018, Han et al. detected low-levels of expression of versican in C3aR^−/−^ ADR mice. They suggested that C3a promoted the transcription of versican. The β-catenin/TCF transcription factor complex was indispensable for the expression of versican. C3a induced the phosphorylation of AKT, which promotes β-catenin/T-cell factor (TCF) expression by inhibiting GSK-3β to directly phosphorylate β-catenin and indirectly stabilize it (45). They treated cultured tubular cells with sera from FSGS patients, which contained high levels of C3a and found that the expression of long non-coding RNA LOC105375913 was increased in a time- and dose-dependent manner, which resulted in the up-regulations of collagen I and fibronectin levels in tubular cells. This expression could be inhibited by a C3aR blocker (46).

Previous studies have shown that C3a/C3aR participates in the pathogenesis of FSGS by regulating various signaling pathways both in glomeruli and tubules, and mentioned the protective function of the C3aR blocker. Furthermore, C3a/C3aR targeted therapy may be applied to FSGS patients.

#### Secondary Glomerular Diseases

##### Lupus nephritis (LN)

LN is mediated by the deposition of immune complexes and complement activation products in the kidney tissue. Studies have shown that both C3a and C3aR are increased in patients with LN and related to disease severity and activity. In 2007, Mizuno et al. found that C3aR staining was positive in 42.9% of all LN kidney specimens and in 81.3% of sections classified as WHO IV LN. The intensity of C3aR staining was positively correlated with LN histological activity score (14). In 2017, Song et al. found that the plasma levels of C3a were elevated, especially in patients with active LN, while they were much lower in patients in remission and in SLE patients without clinical renal involvement (47). Animal experiments have validated the pathogenic effects of C3a/C3aR. As early as in 2005, Bao showed that in the kidneys of MRL/lpr mice, the expression of C3aR was significantly elevated. C3aR antagonist treatment could reduce the expression of IL-1β and RANTES in the kidney, relieve pathological injuries, and prolong survival (48). However, in 2008, Wenderfer found a protective effect of C3aR in the early stage of LN. In C3aR^−/−^MRL/lpr mice at 8 weeks, various pathogenic chemokine receptors were found to be increased, except for MCP-1, and the mice showed an earlier onset of renal injury compared to the controls. However, C3aR expression in glomeruli had no effect on long-term prognosis (49).

Data show high levels of C3a and C3aR expression in LN, but the direct role of C3a/C3aR in the kidney remains to be elucidated. The immune system is over-activated in LN, which reminds us of the role of C3a/C3aR in the immune system mentioned above.

##### Diabetic kidney disease (DKD)

In patients with DKD, C3a/C3aR is remarkably activated and is involved in its pathogenesis. Both the levels of C3a in the plasma and urine were significantly elevated in DKD patients compared to diabetic patients without kidney injury (50, 51). Urinary C3a was positively correlated with urinary protein as well as with the estimated glomerular filtration rate (eGFR). C3a was also correlated with glomerular lesion classification of DKD and the progression of disease (50, 52). C3aR expression was enhanced in early and advanced DKD (53). Li et al. have reported that C3aR antagonist treatment could alleviate kidney damage in DKD rats induced by a high-fat diet and streptozotocin by inhibiting Wnt/β-catenin, TGF-β/smad3 signaling pathways, IKBα phosphorylation, and IL-6 release, to reduce inflammation and fibrosis in glomerular endothelial cells (53, 54). Li et al. have also reported that C3aR-deficiency reduced kidney damage in diabetic rats. The possible mechanism was that the absence of C3aR suppressed T-cell activation by inhibiting the release of cytokines such as IL-4, IL-23, and IL-27 from macrophages (55).

The above studies have clearly demonstrated the pathogenic role of C3a in DKD and suggested its mechanism. The next research direction is to use C3aR as a therapeutic target to treat DKD.

#### Tubulointerstitial Diseases

##### Ischemia-reperfusion (IR) injury

IR injury results in acute tubular necrosis, during which oxygen and nutrients needed to maintain normal metabolism are deprived, where the cells die through necrosis and release abundant endogenous ligands. After restoration of perfusion, endogenous ligands activate innate immune responses by stimulating inflammatory cell recruitment and activation (56, 57). In both tubular epithelial cells and infiltrating neutrophils, monocytes, and macrophages, C3a/C3aR stimulated the production of cytokines and chemokines, such as TNF-α, IFN-γ, MIP-1, MCP-1, IL-1β, IL-6, IL-8, and IL-17, which are thought to be involved in IR kidney injury (56–58). C3a/C3aR also promoted the expression of KIM-1, which is a functional and specific marker for acute tubular necrosis (56, 57). Simone et al. have demonstrated that C3a participated in IR injury by enhancing NADPH oxidase activity and promoting α-SMA protein expression (59). Curci et al. have reported that IR injury promoted the process of C3a-induced epithelial to mesenchymal transition (EMT), which leads to fibrosis through the AKT pathway (60).

The C3a/C3aR pathway promotes ischemia-reperfusion injury through an excess of cytokines and oxidative stress. However, there are few reports on the changes in C3a/C3aR expression. We need to examine whether the expression of C3 is abnormal during the injury. In addition, the direct effect of C3a/C3aR in the kidney remains unresolved and requires further investigation.

##### Chronic tubulointerstitial diseases

Chronic tubulointerstitial inflammation and fibrosis occur in most chronic kidney diseases. Studies have shown that C3a/C3aR can induce tubulointerstitial inflammation and fibrosis by mediating EMT in proximal tubular epithelial cells through the TGF-β1/CTGF signaling pathway (61, 62). The C3aR antagonist effectively inhibited EMT induced by C3a, and ameliorated the pathology of ADR mice and preserved renal function and limited interstitial fibrosis (62). Bao et al. have induced complement activation by transplanting Crry^−/−^C3^−/−^ kidney to C3aR^−/−^ host, where circulating C3 from the host acted on donor kidneys deficient in Crry (a membrane protein that inhibits C3 convertase, equivalent to human CD55) to induce complement activation. The results showed that deficiency in C3aR reduced the kidney tubulointerstitial inflammation and fibrosis (63). C3a could induce T-cells to release IL-17A by ERK, STAT3/5, and NF-κB resulting in an inflammatory response and fibrosis (64).

Although studies have shown the pathogenic effect of C3a/C3aR in chronic tubulointerstitial diseases, its direct renal effect is still unknown. Meanwhile, we cannot confirm the fatal role of the C3a/C3aR pathway in the chronic inflammatory fibrosis process because almost all complement and inflammatory factors are involved in this process. It is worth choosing several important factors for comparison.

#### Urinary Tract Infections

C3a/C3aR exerts a protective effect during infections. For example, in a mouse model of *Listeria monocytogenes*, C3a/C3aR up-regulated Bcl-2 while down-regulated Fas, caspase- 3, and IFN-B, which ameliorate organism-induced apoptosis (65, 66). Similarly, in uropathogenic *Escherichia coli* (UPEC)-induced renal injury, C3a/C3aR also had a protective role by enhancing macrophage phagocytosis induced by LPS despite suppression of inflammatory responses (67, 68). Here, C3a agonism may offer an interesting new therapeutic option along with standard antibiotic care.

## Summary and Prospection

Despite the large number of clinical trials targeting C5, C5a, C5aR1 on AAV, IgAN, aHUS, PNH, and other diseases, and the approved clinical use of eculizumab for PNH, aHUS, and myasthenia gravis, clinical trials targeting C3 are less and mostly on phase I, and no clinical trials targeting C3a or C3aR are registered. Initial discussions primarily relied on theoretical considerations of primary complement deficiencies, whereas C3 deficiency often leads to a broader range of susceptibilities to infections, which is mostly attributed to the opsonic activity of C3b. However, it can be effectively avoided by developing therapies targeting C3a/C3aR, which participates in inflammatory responses but not opsonization. The major impediment derives from the controversies over the C3a/C3aR functions in different cell types and in different diseases, and the complexity of its intercellular signaling pathways. In-depth investigations and awareness of the roles of C3a/C3aR in kidney diseases are needed that will lead to a further expansion of potential indications for complement treatments in the future.

## Author Contributions

SG contributed to analysis and manuscript preparation and wrote the manuscript. ZC helped perform the analysis with constructive discussions. MZ contributed to the conception of the study. All authors contributed to the article and approved the submitted version.

## Conflict of Interest

The authors declare that the research was conducted in the absence of any commercial or financial relationships that could be construed as a potential conflict of interest.
